# Comparison of Peak Oxygen Consumption During Exercise Testing Between Sexes Among Children and Adolescents in Taiwan

**DOI:** 10.3389/fped.2021.657551

**Published:** 2021-05-28

**Authors:** Sheng-Hui Tuan, Guan-Bo Chen, Chia-Hsin Chen, Yi-Jen Chen, I-Hsiu Liou, Yen-Tung Su, Ko-Long Lin

**Affiliations:** ^1^Department of Rehabilitation Medicine, Cishan Hospital, Ministry of Health and Welfare, Kaohsiung, Taiwan; ^2^Department of Physical Therapy, Shu-Zen Junior College of Medicine and Management, Kaohsiung, Taiwan; ^3^Department of Internal Medicine, Kaohsiung Armed Forces General Hospital, Kaohsiung, Taiwan; ^4^Department of Physical Medicine and Rehabilitation, Kaohsiung Medical University Chung-Ho Memorial Hospital, Kaohsiung, Taiwan; ^5^Department of Physical Medicine and Rehabilitation, Kaohsiung Municipal Siaogang Hopsital, Kaohsiung, Taiwan; ^6^Department of Physical Medicine and Rehabilitation, Kaohsiung Veterans General Hospital, Kaohsiung, Taiwan; ^7^School of Medicine, Kaohsiung Medical University, Kaohsiung, Taiwan; ^8^Department of Physical Therapy, Fooyin University, Kaohsiung, Taiwan

**Keywords:** peak oxygen consumption, cardiopulmonary exercise testing, fat-free mass index, sex difference, body mass index

## Abstract

**Objective:** Studies among Western children have observed that the peak oxygen consumption (peak $\dot{\text{V}}$O_2_) of boys is higher than that of girls, and this difference increases as children progress through adolescence. However, the maturation process and social expectation toward Eastern boys and girls are much different from their Western counterparts. This study aimed to provide baseline information on cardiopulmonary fitness (CRF) of Taiwanese children and adolescents in relation to age and sex. We also evaluated the correlation between body mass and CRF and compared the CRF between non-obese and overweight/obese children.

**Methods:** We conducted a retrospective study of children and adolescents aged 4–18 years in Taiwan. Participants were classified into four groups based on age (group 1, aged 4–6; group 2, aged 7–9; group 3, aged 10–13; and group 4, aged 14–18 years). All participants completed symptom-limited exercise test by treadmill and anthropometric measurements through bioelectrical impedance method.

**Results:** In total, 897 (448 men, 449 women) participants were analyzed. Boys had higher peak $\dot{\text{V}}$O_2_ (all *p* < 0.01) and peak metabolic equivalent (MET, all *p* < 0.05) than girls in all the four groups. Age significantly (*P* < 0.001) correlated with peak $\dot{\text{V}}$O_2_ in all participants, boys, and girls, with coefficients of determination (*R*^2^) of 0.9349, 0.9433, and 0.9085, respectively. The peak $\dot{\text{V}}$O_2_ (all *p* < 0.001) of all the groups and peak MET (all *p* < 0.05) of group 2–4 associated with BMI and FMI modestly to moderately. Non-obese children had higher peak MET in group 1 (*p* = 0.049) and group 2–4 (all *p* < 0.001) than overweight/obese children significantly.

**Conclusions:** The difference in peak $\dot{\text{V}}$O_2_ and anthropometry–body composition between sexes was observed earlier in children in Taiwan than those in Western countries. Non-obese children had better CRF than overweight/obese children and the difference presented since preschool age.

## Introduction

The cardiopulmonary exercise testing (CPET) is considered the best method to assess cardiorespiratory fitness (CRF) in adults and children (1, 2), from healthy to ill populations (3, 4). Peak oxygen consumption (peak $\dot{\text{V}}$O_2_), the highest rate at which oxygen can be consumed during exercise, is widely recognized as the best single measure of young people's cardiorespiratory condition (5, 6) since it could provide a composite measure of the pulmonary, cardiovascular, and hematological components of oxygen delivery and oxygen utilization in the exercising muscles.

Many factors, including age and sex, body size and composition, biological, and maturity status, might influence the CRF of young people (7). In general, the peak $\dot{\text{V}}$O_2_ increases in accordance with morphological and physiological changes related to growth and maturation, although the timing and tempo of these changes are specific to each individual (8). There is a near-linear increase in boys' peak $\dot{\text{V}}$O_2_ (L/min) with age and a similar but less consistent trend in girls (9). A longitudinal study in Caucasian children indicates that the absolute peak VO2 is higher in boys than in girls from at least age 10. The same team also observed that the absolute peak VO2 of boys increased with age through the teen years, whereas those of girls appeared to level off from about age 13 (10). A meta-analysis, which selected 20 articles and included 3,808 children, concluded that the peak $\dot{\text{V}}$O_2_ for prepubertal boys is 18% higher than those in girls (5). However, many studies about the peak $\dot{\text{V}}$O_2_ of children and adolescents have been performed in Western countries. The peak $\dot{\text{V}}$O_2_ has been shown to vary by ethnic group and is consistently lower in Chinese adults than in Caucasian adults (11, 12). The differences in the peak $\dot{\text{V}}$O_2_, though not well-understood, might be related to environmental, socio-ecological factors at the individual, family, school and cultural levels or developmental discrepancy from one race to another (13, 14). There is also a variation in the peak $\dot{\text{V}}$O_2_ in children of different races or those from different residential regions (15). Given the sparse data of Southern Chinese children, a study in Hong Kong found that the absolute peak $\dot{\text{V}}$O_2_ values for Chinese girls and boys aged <10 years were 17% and 19%, respectively, which are lower than the predicted values in Caucasian children, but were comparable with values in Caucasian adolescents (16).

One of the possible explanation for the difference of peak $\dot{\text{V}}$O_2_ might be the different age of reaching peak height velocity. Children who reach peak height velocity at an earlier age have less time available for prepubertal growth. This may have significant impact on the development of oxygen uptake, given that it is highly correlated with body (16). The decline in age at puberty in the general population has been noted in both sexes based on worldwide data (17–19). Age at menarche (AAM) is an important event in a woman's life and is determined by both biological and environmental factors, such as body weight, nutrition, genetics, and socioeconomic status (20). From the 1960s, the trend in AAM appears to have leveled off significantly both in the UK and USA at 2.5–4 months during the past 25 years (17). The mean AAM declined by 0.42 years/decade in women born in the period from 1955 to 1985 in southeast China (19). With respect to boys, the Copenhagen Puberty Study reported a decline of 3 months in the age at onset of puberty during a recent 15-year period (21). Different developmental stages might affect the oxygen transport system and results of CPET in children. The present discrepancy in the peak $\dot{\text{V}}$O_2_ between sexes and races in children might be different from that in the past.

Many studies have proved that children or adolescents with obesity have lower CRF, which negatively affected the cardiovascular system (7, 22). Studies from different countries and races have shown that the physical fitness of young people correlates negatively with increasing body fat mass (10, 23, 24). A higher body mass index (BMI) and increased percentage of fat mass had been proved to be negatively related with the CRF level in children and adolescents with normal weight or overweight status (7, 25, 26). Our team used CPET to measure the peak $\dot{\text{V}}$O_2_ directly and found that excessive body adiposity, regardless of BMI or fat mass index (FMI), negatively affected the CRF of schoolchildren aged 10–18 years (1). However, we did not analyze the correlation between body composition and CRF at different ages nor in children aged <10 years. The inclusion of different ages and appropriate adjustment for body size are crucial; however, to date, only few studies have appropriately documented baseline values of peak $\dot{\text{V}}$O_2_ from preschool to schoolchildren in Taiwan. Thus, the present study aimed to enhance the understanding of (1) the development of peak $\dot{\text{V}}$O_2_ at aged 4–18 years in relation to sex, age groups, and body composition of Taiwanese children and (2) to evaluate the discrepancy in peak $\dot{\text{V}}$O_2_ between Taiwanese and Western children and between Taiwanese boys and girls.

## Methods and Materials

### Subject Characteristics

This retrospective study was conducted at a tertiary Medical Center in southern Taiwan from July 2013 to July 2019. All preschool children, schoolchildren, and adolescents (aged 4–18 years) without known significant medical conditions and detectable cardiovascular disease (examined by 12-lead electrocardiogram and transthoracic echocardiography) were recruited. A physiatrist specialized in CPET with more than 15 years of experience (K.L.L) reviewed all CEPT data, and participants who could not reach peak effort during exercise testing were excluded. The peak $\dot{\text{V}}$O_2_ was determined when two of the following three conditions were met: (1) respiratory exchange ratio (RER) >1.0, (2) heart rate (HR) within 5% of the age-predicted maximum, (3) and the participant was exhausted and refused to continue despite strong verbal encouragement (27). Before enrollment, each participant was familiarized with the procedures and equipment used in the treadmill exercise testing through a demonstrative explanation. The purpose of the study was explained to the participants and their families before written informed consent was obtained. Participants were classified into the following four groups based on age: group 1, age 4–6 years; group 2, aged 7–9 years; group 3, aged 10–13 years; group 4, 14–18 years. A fifth group (group 5) was added, which was composed of all children and adolescents (aged 4–18 years).

This study was conducted in accordance with the Helsinki Declaration and was approved by the Institutional Review Board of Kaohsiung Veterans General Hospital (number: VGHKS15-CT7-05).

### Anthropometry-Body Composition

Height and weight of barefooted participants wearing light clothing were measured during visit. All measurements were taken by trained physical therapist following standard operating procedures. The anthropometry–body composition was measured by vector bioelectrical impedance analysis (VBIA), which is a useful tool for body composition analysis in healthy adults and children. The VBIA was performed with bioelectrical impedance vector analysis software by the resistance-reactance graph method (28). To analyze the body composition of the participants, Zeus 9.9 PLUS (Jawon Medical Co., Ltd., Kungsang Bukdo, South Korea) was used, which sent a minute electrical current and measured the body composition using personal data that had already been saved (height, weight, sex, age, and newly calculated body impedance) by the tetrapolar electrode method; the electrodes were located on both hands, both soles, and both ankles, with frequency of 1, 5, 50, 250, 550, and 1,000 kHz and current of 360 uA.

BMI was calculated by dividing the weight (kg) by the square of the participant's height (cm). Children and adolescents were categorized as “underweight,” “normal weight,” “overweight,” and “obese,” using standard age- and gender-specific BMI values published in 2013 by the Ministry of Education of Taiwan (29). The FMI was defined as fat mass (kg) divided by the square (m^2^) of the participant's height, and the fat-free mass index (FFMI) was defined as fat-free mass (kg) divided by the square (m^2^) of the participant's height.

### Treadmill Exercise Testing

To measure the participants' exercise capacity, a graded symptom-limited exercise testing system was employed, which was composed of a treadmill, a flow module, a gas analyzer, and an electrocardiographic monitor (Metamax 3B, Cortex Biophysik GmbH Co., Germany). All participants completed the testing according to the Bruce ramp protocol suggested by the American College of Sports Medicine. The test was terminated when the preschoolers demonstrated subjective unbearable symptoms or when they could no longer continue the testing (30). The metabolic equivalent (MET), blood pressure, and HR were measured throughout the testing. MET at anaerobic threshold (AT MET) was also recorded. The AT was determined by the VE/$\dot{\text{V}}$O_2_ and VE/VCO_2_ methods (31). The peak $\dot{\text{V}}$O_2_ was the maximum oxygen uptake measured at peak exercise, and the peak MET was calculated as peak $\dot{\text{V}}$O_2_ divided by 3.5 ml kg^−1^ min^−1^. Peak exercise was determined when two of the following three conditions were met: (1) RER > 1.0, (2) HR within 5% of the age-predicted maximum, and (3) the participant was exhausted and refused to continue the test despite strong verbal encouragement (27). The peak $\dot{\text{V}}$O_2_ to the predicted value (peak PD) was the percentage of the measured peak MET to the predicted peak MET based on the study by Armstrong (32).

### Statistical Analysis

SPSS for Windows version 19.0 (IBM Corp., Armonk, NY) was used for all analyses. Continuous data were expressed as mean ± standard deviation, and categorical variables were presented as absolute numbers or percentages. Normality and homoscedasticity were checked prior to each analysis. With respect to the comparison of data between sexes and children from different BMI groups, the independent *t*-test was used for normally distributed variables, while the Mann–Whitney U test was used for non-normally distributed variables. Comparison of aerobic fitness in relation to age was assessed using one-way analysis of variance (ANOVA), and a *post-hoc* test was performed by Scheffe method if there was homogeneity of variance or the Dunnett T3 test if there was heterogeneity of variance. Correlations of BMI, FMI, and aerobic fitness (AT MET, peak MET, peak $\dot{\text{V}}$O_2_, and peak PD) of all subjects were examined using Pearson's correlation analysis for normally distributed variables and Spearman's correlation analysis for non-normally distributed variables. A *P*-value ≤ 0.05 was considered significant.

## Results

A total of 992 data of CEPT were collected initially, of which 16 (1.6%) were excluded from the study owing to incomplete electrocardiogram and transthoracic echocardiographic data. Another 79 (8.8%) patients who failed to meet the criteria for a maximal effort were excluded. Finally, data from 897 (448 male, 449 female) children and adolescents were included in the analysis. Among them, 80 (44 male, 36 female), 163 (90 male, 73 female), 342 (169 male, 173 female), and 312 (145 male, 167 female) participants were allocated to groups 1, 2, 3, and 4, respectively. Descriptive data for each sex of these four groups are shown in Table 1. Sex differences in FFM and FFMI were significant in all four groups. While boys had higher weight and height than girls in the two oldest groups, a significant difference in FMI and BMI between sexes was observed in groups 2–4 and group 3, respectively (Table 1).

**Table 1:** Comparison of body composition between two sexes in relation to age group.

**Age group**		**Height**	**body weight**	**BMI**	**BMI group**				**Fat mass**	**Fat-free mass**	**FMI**	**FFMI**
					**Underweight**	**Normal weight**	**Overweight**	**Obese**				
Age 4–6	Boys (*n* = 44)	119.04 ± 5.72	22.76 ± 5.22	16.01 ± 3.30	8 (18.2%)	29 (65.9%)	4 (9.1%)	3 (6.8%)	2.80 ± 0.54	3.92 ± 0.75	1.99 ± 0.38	12.26 ± 0.43
	Girls (*n* = 36)	117.00 ± 4.10	20.74 ± 3.37	15.12 ± 2.19	12 (33.3%)	20 (55.5%)	2 (5.6%)	2 (5.6%)	3.46 ± 2.23	17.89 ± 1.55	2.47 ± 1.60	12.93 ± 1.01
	*P*-value	0.078	0.048^*^	0.167	N/A				0.717	<0.001^*^	0.607	<0.001^*^
Age 7–9	Boys (*n* = 90)	128.86 ± 8.00	30.01 ± 7.78	17.90 ± 3.37	12 (13.3%)	56 (62.3%)	13 (14.4%)	9 (10.0%)	4.32 ± 3.44	26.03 ± 5.01	2.55 ± 1.89	15.46 ± 1.78
	Girls (*n* = 73)	127.28 ± 8.05	28.27 ± 7.73	17.53 ± 6.05	10 (13.7%)	50 (68.5%)	8 (10.9%)	5 (6.9%)	6.08 ± 4.01	22.63 ± 4.59	3.72 ± 2.43	13.86 ± 1.93
	*P*-value	0.215	0.158	0.621	N/A				0.004^*^	<0.001^*^	0.001^*^	<0.001^*^
Age 10–13	Boys (*n* = 169)	151.64 ± 11.39	46.15 ± 13.33	20.32 ± 8.93	27 (16.0%)	93 (55.0%)	26 (15.4%)	23 (13.6%)	7.96 ± 6.19	38.91 ± 8.38	3.37 ± 2.42	16.65 ± 2.00
	Girls (*n* = 173)	147.20 ± 11.25	40.56 ± 10.16	18.42 ± 3.57	37 (21.4%)	108 (62.4%)	17 (9.8%)	11 (6.4%)	8.69 ± 4.61	31.85 ± 6.44	4.01 ± 2.06	14.58 ± 1.67
	*P*-value	<0.001	<0.001	0.013^*^	N/A				0.184	<0.001^*^	0.008^*^	<0.001^*^
Age 14–18	Boys (*n* = 145)	170.33 ± 7.51	62.29 ± 17.31	21.33 ± 5.08	31 (21.4%)	75 (51.7%)	22 (15.2%)	17 (11.7%)	11.95 ± 9.68	50.36 ± 8.61	4.05 ± 3.15	17.29 ± 2.21
	Girls (*n* = 167)	158.14 ± 5.53	51.83 ± 9.36	20.62 ± 3.70	28 (16.8%)	112 (67.1%)	14 (8.4%)	13 (7.7%)	13.43 ± 5.78	38.59 ± 4.20	5.38 ± 2.29	15.42 ± 1.42
	*P*-value	<0.001	<0.001	0.157	N/A				0.100	<0.001^*^	<0.001^*^	<0.001^*^

*BMI, body mass index; FMI, fat mass index; FFMI, fat-free mass index; N/A, non-applicable*.

*P-values, comparisons between boys and girls*.

* *p < 0.05*.

**Figure 1:**
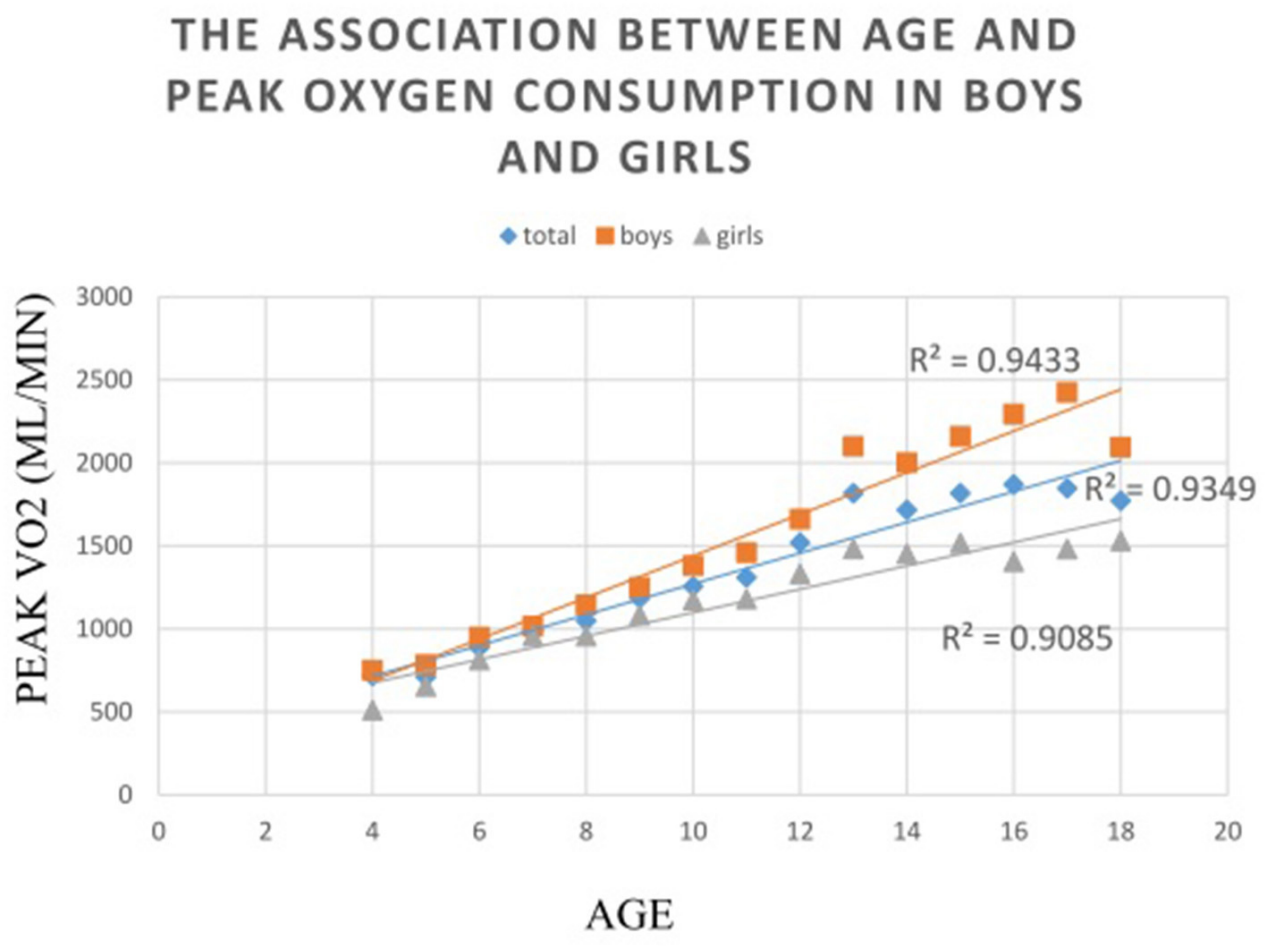
The associations between absolute peak oxygen consumption to sex in relation to age. The relationships between absolute peak oxygen consumption to age are illustrated in this figure. Age significantly (*P* < 0.001) correlated with peak VO2 in all participants, boys, and girls, with coefficients of determination (*R*^2^) of 0.9349, 0.9433, and 0.9085, respectively.

As regards the comparison between sexes, boys had significantly higher AT MET, peak MET and peak $\dot{\text{V}}$O_2_ and lower peak PD than girls from the three older groups (all *P* < 0.01). Significant differences were also found in the peak MET (*P* = 0.022), peak $\dot{\text{V}}$O_2_ (*P* = 0.001), and peak PD (*P* < 0.001) between the two sexes in group 1. Results of an ANOVA test showed that AT MET, peak MET, and peak $\dot{\text{V}}$O_2_ were significantly related to age in both sexes (all *P* < 0.01). *Post-hoc* tests confirmed a significant difference in the absolute peak $\dot{\text{V}}$O_2_ between each group in each sex. As regards $\dot{\text{V}}$O_2_ in relation to mass, significant differences were apparent only between the two older groups for either sex in the analysis of AT MET and peak MET. The absolute peak $\dot{\text{V}}$O_2_ values for boys and girls were 26.61–29.38% and 8.58–21.92%, respectively, which are lower than the predicted values for Caucasians. Results of an ANOVA test showed that the peak PD was significantly related to age only in girls but not in boys. The *post-hoc* analysis revealed no significant difference between the two younger age groups (Table 2). The relationships between absolute peak $\dot{\text{V}}$O_2_ and age according to sex are illustrated in Figure 1. Age significantly (*P* < 0.001) correlated with peak $\dot{\text{V}}$O_2_ in all participants, boys, and girls, with coefficients of determination (R^2^) of 0.9349, 0.9433, and 0.9085, respectively.

**Table 2:** Comparison of cardiopulmonary fitness during exercise testing between two sexes in relation to age group.

**Age group**		**AT MET** ** (kcal/kg/hour)**	**Peak MET** ** (kcal/kg/hour)**	**Peak $\dot{\text{V}}$O_**2**_** ** (ml/min)**	**Peak PD** ** (%)**
Group 1 age 4–6	Boys (*n* = 44)	8.52 ± 1.47^b,c^	11.40 ± 1.79^c^	896.0 ± 183.2^a−c^	73.39 ± 12.13
	Girls (*n* = 36)	7.96 ± 1.39^b,c^	10.54 ± 1.47^b,c^	764.7 ± 160.1^a−c^	91.42 ± 14.16^b,c^
	*P*-value	0.088	0.022^*^	0.001^*^	<0.001^*^
Group 2 Age 7–9	Boys (*n* = 90)	8.02 ± 1.43^d,e^	11.23 ± 1.78^e^	1148.4 ± 206.9^a,d−e^	71.67 ± 13.81
	Girls (*n* = 73)	7.45 ± 1.28^d,e^	10.30 ± 1.55^d,e^	1001.1 ± 225.6^a,d,e^	91.39 ± 13.67^d,e^
	*P*-value	0.009^*^	0.001^*^	<0.001^*^	<0.001^*^
Group 3 Age 10–13	Boys (*n* = 169)	7.27 ± 1.39^b,d^	10.57 ± 2.00	1671.4 ± 472.2^b,d,f^	71.12 ± 16.21
	Girls (*n* = 173)	6.56 ± 1.17^b,d,f^	9.13 ± 1.48^b,d,f^	1276.8 ± 309.4^b,d,f^	83.22 ± 13.29^b,d,f^
	*P*-value	<0.001^*^	<0.001^*^	<0.001^*^	<0.001^*^
Group 4 Age 14–18	Boys (*n* = 145)	6.93 ± 1.39^c,e^	10.23 ± 1.98	2190.2 ± 626.6^c,e,f^	70.62 ± 13.60
	Girls (*n* = 167)	5.82 ± 1.03^c,e,f^	8.17 ± 1.33^c,e,f^	1472.9 ± 303.6^c,e,f^	78.08 ± 12.04^c,e,f^
	*P*-value	<0.001^*^	<0.001^*^	<0.001^*^	<0.001^*^
ANOVA	Boys	*F*_(3, 444)_ = 20.920, *p* < 0.008^*^	*F*_(3, 444)_ = 7.220*, p < * 0.001^*^	*F*_(3, 444)_ = 134.230, *p < * 0.001^*^	*F*_(3, 445)_ = 0.438, *p* = 0.726
	Girls	*F*_(3, 445)_*=* 54.167, *p < * 0.001^*^	*F*_(3, 445)_ = 52.314, *p < * 0.001^*^	*F*_(3, 445)_ = 87.171, *p < * 0.001^*^	*F*_(3, 445)_ = 23.175*, p < * 0.001^*^

*Data was presented as mean ± standard deviation; AT MET, metabolic equivalent at anaerobic threshold; ANOVA, one-way analysis of variance in each sex; Peak MET, peak metabolic equivalent; Peak $\dot{\text{V}}$O_2_, peak oxygen consumption; Peak PD, peak $\dot{\text{V}}$O_2_ to predicted value*.

*P-values, comparisons between boys and girls*.

a *Post-hoc test showed significantly different data between group 1 and group 2*.

b *Post-hoc test showed significantly different data between group 1 and group 3*.

c *Post-hoc test showed significantly different data between group 1 and group 4*.

d *Post-hoc test showed significantly different data between group 2 and group 3*.

e *Post-hoc test showed significantly different data between group 2 and group 4*.

f *Post-hoc test showed significantly different data between group 3 and group 4*.

* *p < 0.05*.

Table 3 demonstrates the correlation between BMI, FMI, and performance of exercise test of children and adolescents. The peak $\dot{\text{V}}$O_2_ was significantly positively associated with BMI and FMI in all the groups. The correlation coefficient ranged from 0.177 to 0.659. All showed modest to moderate positive correlations. The peak MET and peak PD were significantly negatively associated with BMI and FMI of participants from groups 2, 3, 4, and 5. The correlation coefficient ranged from −0.160 to −0.674. All showed modest to moderate negative correlations. Associations of peak exercise test performance with BMI or FMI were significantly negative in group 1, but not for associations of (1) peak MET with BMI or FMI in girls and (2) peak MET with BMI in both sexes in group 1. AT MET significantly negatively correlated with BMI and FMI in the participants of groups 2–5. The correlation coefficient was between −0.110 and −0.606. All showed modest to moderate negative correlations. In group 1, no significant association was found between AT MET and BMI or FMI, except between AT MET and BMI in boys.

**Table 3:** Correlation between body mass index, fat mass index, and performance of exercise test of children and adolescents.

**Age group**			**AT MET** ** (kcal/kg/hour)**	**Peak MET** ** (kcal/kg/hour)**	**Peak $\dot{\text{V}}$O_**2**_** ** (ml/min)**	**Peak PD** ** (%)**
Group 1 Age 4–6	Boys (*n* = 44)	BMI FMI	−0.317^*^ (0.036) −0.257 (0.195)	−0.352^*^ (0.019) −0.407^*^ (0.035)	0.659^**^ (<0.001) 0.549^*^ (0.003)	−0.262 (0.085) −0.303 (0.125)
	Girls (*n* = 36)	BMI	0.032 (0.854)	−0.060 (0.729)	0.638^**^ (<0.001)	−0.033 (0.850)
		FMI	−0.043 (0.826)	−0.143 (0.460)	0.593^**^ (0.001)	−0.138 (0.476)
	Total (*n* = 80)	BMI	−0.160 (0.156)	−0.211 (0.061)	0.652^**^ (<0.001)	−0.225^*^ (0.045)
		FMI	−0.177 (0.192)	−0.306^*^ (0.022)	0.486^**^ (<0.001)	−0.132 (0.334)
Group 2 Age 7-9	Boys (*n* = 90)	BMI	−0.606^**^ (<0.001)	−0.674^**^ (<0.001)	0.431^**^ (<0.001)	−0.533^**^ (<0.001)
		FMI	−0.536^**^ (<0.001)	−0.613^**^ (<0.001)	0.281^*^ (0.008)	−0.473^**^ (<0.001)
	Girls (*n* = 73)	BMI	−0.141 (0.232)	−0.310^*^ (0.010)	0.422^**^ (<0.001)	−0.295^*^ (0.011)
		FMI	−0.261^*^ (0.034)	−0.418^**^ (<0.001)	0.540^**^ (<0.001)	−0.418^**^ (<0.001)
	Total (*n* = 163)	BMI	−0.309^**^ (<0.001)	−0.411^**^ (<0.001)	0.403^**^ (<0.001)	−0.328^**^ (<0.001)
		FMI	−0.430^**^ (<0.001)	−0.544^**^ (<0.001)	0.301^**^ (<0.001)	−0.191^*^ (0.018)
Group 3 Age 10–13	Boys (*n* = 169)	BMI	−0.140 (0.071)	−0.213^*^ (0.005)	0.177^*^ (0.022)	−0.205^**^ (0.008)
		FMI	−0.388^**^ (<0.001)	−0.525^**^ (<0.001)	0.316^**^ (<0.001)	−0.486^**^ (<0.001)
	Girls (*n* = 173)	BMI	−0.141 (0.232)	−0.301^*^ (0.010)	0.422^**^ (<0.001)	−0.295^*^ (0.011)
		FMI	−0.261^*^ (0.034)	−0.418 ^**^(<0.001)	0.540^**^ (<0.001)	−0.418^**^ (<0.001)
	Total (*n* = 342)	BMI	−0.110^*^ (0.043)	−0.160^*^ (0.003)	0.279^**^ (<0.001)	−0.252^**^ (<0.001)
		FMI	−0.329^**^ (<0.001)	−0.460^**^ (<0.001)	0.241^**^ (<0.001)	−0.318^**^ (<0.001)
Group 4 Age 14–18	Boys (*n* = 145)	BMI	−0.419^**^ (<0.001)	−0.438^**^ (<0.001)	0.447^**^ (<0.001)	−0.445^**^ (<0.001)
		FMI	−0.451^**^ (<0.001)	−0.470^**^ (<0.001)	0.437^**^ (<0.001)	−0.472^**^ (<0.001)
	Girls (*n* = 167)	BMI	−0.125 (0.107)	−0.191^*^ (0.013)	0.539^**^(<0.001)	−0.180^*^ (0.020)
		FMI	−0.195^*^ (0.012)	−0.279^**^ (<0.001)	0.460^**^ (<0.001)	−0.260^*^ (0.001)
	Total (*n* = 312)	BMI	−0.244^**^ (<0.001)	−0.253^**^ (<0.001)	0.420^**^ (<0.001)	−0.339^**^ (<0.001)
		FMI	−0.410^**^ (<0.001)	−0.456^**^ (<0.001)	0.201^**^ (<0.001)	−0.280^**^ (<0.001)
Total Age 4–18	Boys (*n* = 448)	BMI	−0.325^**^ (<0.001)	−0.348^**^ (<0.001)	0.365^**^ (<0.001)	−0.292^**^ (<0.001)
		FMI	−0.462^**^ (<0.001)	−0.524^**^ (<0.001)	0.432^**^ (<0.001)	−0.453^**^ (<0.001)
	Girl (*n* = 449)	BMI	−0.308^**^ (<0.001)	−0.384^**^ (<0.001)	0.578^**^ (<0.001)	−0.334^**^ (<0.001)
		FMI	−0.336^**^ (<0.001)	−0.423^**^ (<0.001)	0.547^**^ (<0.001)	−0.378^**^ (<0.001)
	Total (*n* = 879)	BMI	−0.276^**^ (<0.001)	−0.295^**^ (<0.001)	0.419^**^ (<0.001)	−0.308^**^ (<0.001)
		FMI	−0.439^**^ (<0.001)	−0.512^**^ (<0.001)	0.338^**^ (<0.001)	−0.296^**^ (<0.001)

*Data was presented as Pearson's coefficient factor (p-value)*.

*AT MET, metabolic equivalent at anaerobic threshold; Peak MET, peak metabolic equivalent; Peak $\dot{\text{V}}$O_2_, peak oxygen consumption; Peak PD, peak $\dot{\text{V}}$O_2_ to predicted value*.

* *p-value < 0.05*,

** *p-value < 0.001*.

**Figure 2:**
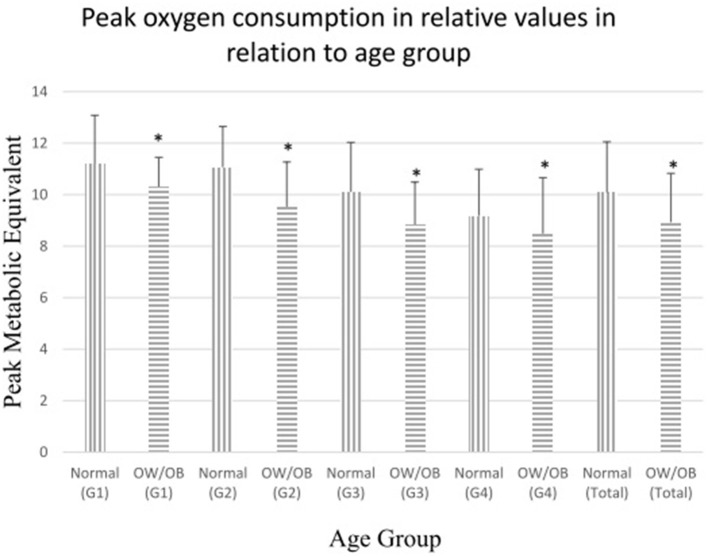
Comparisons of peak oxygen consumption in relative values (metabolic equivalent) between children with normal and overweight/obese body mass index. Peak oxygen consumption in relative values (metabolic equivalent) in normal-weight/non-obese and overweight/obese children. OW/OB = overweight/obese children. G1: children aged 4–6, G2: children aged 7–9, G3: children aged 10–13, G4: children aged 144–18, Total: children aged 4–18. *p < 0.05.

Table 4 presents the results of the comparison of cardiopulmonary fitness during CPET between children and adolescents with normal and overweight/obese status in relation to the age group. In all groups, participants with normal weight status had better AT MET, peak MET, and peak PD, except that no significant difference was found in the AT MET and peak PD in group 1 (Figure 2).

**Table 4:** Comparison of cardiopulmonary fitness during exercise testing between children with normal and overweight/obese body mass index in relation to age group.

**Age group**		**AT MET** ** (kcal/kg/hour)**	**Peak MET** ** (kcal/kg/hour)**	**Peak PD** ** (%)**
Group 1 Age 4–6	Normal BMI (*n* = 49)	8.44 ± 1.55	11.21 ± 1.87	81.32 ± 16.07
	OW/OB BMI (*n* = 11)	7.81 ± 0.92	10.31 ± 1.14	77.13 ± 16.91
	*P*-value	0.088	0.049^*^	0.442
Group 2 Age 7–9	Normal BMI (*n* = 116)	7.90 ± 1.31	11.07 ± 1.58	82.69 ± 16.61
	OW/OB BMI (*n* = 35)	6.84 ± 1.28	9.53 ± 1.74	69.72 ± 15.16
	*P*-value	<0.001^**^	<0.001^**^	<0.001^**^
Group 3 Age 10–13	Normal BMI (*n* = 201)	7.06 ± 1.30	10.11 ± 1.92	79.47 ± 12.87
	OW/OB BMI (*n* = 77)	6.40 ± 1.18	8.84 ± 1.65	66.17 ± 14.16
	*P*-value	<0.001^**^	<0.001^**^	<0.001^**^
Group 4 Age 14–18	Normal BMI (*n* = 187)	6.33 ± 1.23	9.18 ± 1.81	76.55 ± 12.08
	OW/OB BMI (*n* = 66)	5.89 ± 1.31	8.49 ± 2.17	66.63 ± 15.08
	*P*-value	0.014^*^	0.012^*^	<0.001^**^
Total Age 4–18	Normal BMI (*n* = 553	7.10 ± 1.48	10.08 ± 1.97	79.26 ± 13.89
	OW/OB BMI (*n* = 189)	6.39 ± 1.32	8.93 ± 1.90	67.63 ± 14.96
	*P*-value	<0.001^**^	<0.001^**^	<0.001^**^

*Data was presented as mean ± standard deviation; AT MET, metabolic equivalent at anaerobic threshold; Peak MET, peak metabolic equivalent; Peak PD, peak oxygen consumption to predicted value, BMI, body mass index; OW/OB, overweight/obese; P-value s, comparisons between Normal and OW/OB BMI*;

* *P-value < 0.05*,

** *p-value < 0.001*.

## Discussion

Our data show that absolute peak $\dot{\text{V}}$O_2_ values for Taiwanese children and adolescents are considerably lower than those of their Caucasian peers. Moreover, the absolute peak $\dot{\text{V}}$O_2_ values, but not the peak $\dot{\text{V}}$O_2_ in relation to mass, increase with age in both sexes. Children with normal weight had better CRF than those overweight or obese states and the trend presented since preschool age. The discrepancy in both absolute peak $\dot{\text{V}}$O_2_ and peak MET as well as FFM and FFMI between sexes, much to our surprise, presented earlier in preschool age.

In this study, the absolute peak $\dot{\text{V}}$O_2_ increase with age in both sexes in a perfect linear pattern, similar to those in previous reports (27, 33). We could observe a near-linear increase in absolute peak $\dot{\text{V}}$O_2_ with aging in boys. As the rate of growth change decreased between age 16 and 18 years, the age effect was reduced. Moreover, we found a near-linear increase in the absolute peak $\dot{\text{V}}$O_2_ in girls until from age 13 to 15 years, followed by a leveling off of values. This trend of increasing peak $\dot{\text{V}}$O_2_ in relation to age was similar with those found by previous cross-sectional and longitudinal studies (10). Sex differences in the development of CRF might have contributed to the differences in physical activity (13), maximal stroke volume, and maximal arteriovenous oxygen difference (34).

In this study, the absolute peak $\dot{\text{V}}$O_2_ values are considerably lower than the predicted values based on Armstrong's regression equations (32), which were generated from data on Caucasian children and adolescents. Since Taiwanese children reach peak height velocity at an earlier age than Caucasian children, there is possible less time for prepubertal growth and therefore developmentally divergent peak $\dot{\text{V}}$O_2_ (35). The difference in the peak $\dot{\text{V}}$O_2_ between Taiwanese and Western children might be multifactorial and possibly related to the following factors:

(1) Smaller body size of Taiwanese children. Although the stature difference between Taiwanese and Western children decreased gradually in the last decade, the median height according to the World Health Organization (WHO) standard is higher by an average of 0.1 cm for both boys and girls aged 0–5 years, 0.5 cm for boys and 1.0 cm for girls aged 7–13 years, and 2.5 cm for boys and 2.9 cm for girls aged 13–18 years (36).

(2) Nutrition. In the nutrition and health survey of Taiwanese elementary school children, cereal foods are staple and animal protein is relatively insufficient (37).

(3) Small amount of time for outdoor activities and exercise. According to the 2018 report card on physical activity for children and youth in Taiwan, respondents scored very poor on the overall physical activity and organized sport participation dimensions (38). Based on the nutrition and health survey in Taiwan, only 12.1% of Taiwanese adolescents aged 15–18 years and 5.4% of 13–15 years met the WHO standard of at least 60 min of moderate to vigorous physical activity per day. Moreover, only 5.8 and 2.8% of boys and girls aged 7–12 years, respectively, engaged in moderate physical activity more than four times a week (38).

In this study, we observed that the discrepancy in the absolute peak $\dot{\text{V}}$O_2_ between Taiwanese and Caucasian children and adolescents still presented after aged 13 years in both sexes and the gap even became larger with aging among girls. Using Armstrong's regression equations, McManus et al. observed that values are 18% and 22%, and 32% and 22% lower than predicted for boys and girls aged <10 years and 10–13 years in Hong Kong, respectively. The measured absolute peak $\dot{\text{V}}$O_2_ for the 13–16 age group was only 3% lower than predicted for either sex (16). Our data are different from their findings. The decline in the motor ability of Asian girls is related to the physical, physiological, and perhaps behavioral changes during adolescence (39, 40) which results in the larger gap between different races. By contrast, boys participated more in sports during adolescence, which results in greater muscle mass and the discrepancy of peak $\dot{\text{V}}$O_2_ remained (38, 41).

We observed a greater peak $\dot{\text{V}}$O_2_ in boys than in girls, with the difference gradually widening as age increases. These findings were similar with those of previous studies (8, 16, 27). This sex-related variability in peak $\dot{\text{V}}$O_2_ might relate mainly to the differences in body composition owing to the marked increase in FFMI after puberty in boys (10). Boys have a higher ratio of FFMI and a lower ratio of total body fat/stature^2^ after adolescence (42). In the present study, the lower peak $\dot{\text{V}}$O_2_ in girls compared with boys after age 13 years may be also related to the onset of menarche at around age 13 years (seventh grade) in girls in Taiwan (43), which results in increases in fat mass and reduction in growth rate (44). Armstrong et al. observed that the FFM increases by ~40 and 90% in girls and boys, respectively, from age 11 to 16 years (8). One study found that boys have a sex-specific increase in hemoglobin concentration in the late teenage years, which enhances the oxygen-carrying capacity of the blood (45).

We found an earlier significant discrepancy in body composition and peak $\dot{\text{V}}$O_2_ between sexes in the prepubertal stage, even in the preschool stage. This finding contradicts with those of previous studies (16, 27). This difference might be caused by multiple factors. Studies using Doppler echocardiography have indicated that boys have greater stroke volume (>10%) than girls, which could partially account for the small prepubertal sex difference in peak $\dot{\text{V}}$O_2_ (46). A study using near-infrared spectroscopy also reported poorer matching of muscle oxygen delivery to oxygen utilization in prepubertal girls compared with boys and concluded that this difference may contribute to sex differences in peak oxygen uptake (47). FFM has a powerful influence on $\dot{\text{V}}$O_2_ in children regardless of sexes (10). Our previous study also showed that preschoolers with higher FFMI had better peak $\dot{\text{V}}$O_2_ during treadmill exercise testing (48). Since boys had higher FFMI than girls in all four groups, it is reasonable to find the discrepancy in peak $\dot{\text{V}}$O_2_ between sexes. The differences in body composition between boys and girls could be partially explained by the decline in age at puberty nowadays, which could cause early maturation (17, 21). Many of the aforementioned effects of maturity might have interfered the results of maximal effort during CPET (41, 45). However, precocious puberty could not account for the differences in the body composition and peak $\dot{\text{V}}$O_2_ observed in preschool children. Many biological and environmental factors, such as nutrition status, different social expectations toward boys and girls in the Taiwanese society, might contribute to that finding.

In this study, children with normal weight had significantly better CRF than those with overweight or obese status, and the difference presented in early childhood since age 4. Our findings were consistent with those of previous studies (23–25); in addition, we included data measured directly from CPET of preschoolers aged 4–6 years. Childhood obesity is now recognized as a serious public health concern. The increase in the prevalence of childhood obesity has been documented as early as the preschool years, and the global prevalence of preschool overweight and obesity escalated to 9.1% in 2020 (49). Besides the lowering of the CRF in this study, preschool obesity causes serious health consequences in both physical and psychological aspects in the long term (50). Childhood overweight and obesity should be prevented as early as possible.

This study has several limitations. First, this study was conducted in a single Medical Center in southern Taiwan, and the results might be only generalized to similar populations, even though the basic characteristics of the recruited participants were similar to the data found in the national survey in Taiwan (36). Our study presented data only for a limited subpopulation of healthy Taiwan children and adolescents; hence, these preliminary results should be carefully applied to the entire population. Second, since only bioelectrical impedance data about body composition were collected, we did not include lean body mass (51). Third, CPET was performed on a treadmill, and the results would not be applicable if the test was performed using other ergometers such as a cycle ergometer (52). Fourth, data were categorized into four age groups. We did not use a scaling approach age-by-age to demonstrate the difference in the peak exercise capacity between sexes at specific age owing to the relative small sample numbers.

As study strengths, we measured the CRF of young participants aged 4–18 years directly by graded symptom-limited treadmill exercise testing with a gas analyzer rather than by other indirect measurements, such as the 20-m multistage fitness test (53) and 800- meter run (54) that are used to reflect CRF of Taiwanese or Chinese children. In this study, data might more accurately reflect the CRF of the participants by directly acquiring the peak $\dot{\text{V}}$O_2_. To our knowledge, this is the first study to provide data of peak $\dot{\text{V}}$O_2_ from age 4 to 18 years in relation to sex, age groups, and body composition of Taiwanese children. In addition, our results enhanced the importance of health promotion and weight control programs as early as in preschool age to prevent childhood obesity.

## Conclusions

This study provided descriptive data of the development of peak $\dot{\text{V}}$O_2_ from age 4 to 18 years in relation to sex. The results of this study showed that Taiwanese children and adolescents exhibited a distinct pattern of development relative to Caucasian children. The discrepancy in peak $\dot{\text{V}}$O_2_ between sexes was noted earlier since preschool age. Further large-scale and nationwide studies are warranted to provide a scaling approach age-by-age or by objective measure of maturation status such as the Tanner stage among Taiwanese children and adolescents to clarify this earlier discrepancy of peak exercise capacity. Moreover, we found that the significant difference in the CRF between children with normal weight and overweight/obese status presented since preschool age. Given the high prevalence of excessive adipose in children and adolescents, weight control, and health promotion are important for improving public health, which should be started as early as possible.

## Data Availability Statement

The raw data supporting the conclusions of this article will be made available by the authors, without undue reservation.

## Ethics Statement

This study was conducted in accordance with the Helsinki Declaration and was approved by the Institutional Review Board of Kaohsiung Veterans General Hospital (number: VGHKS15-CT7-05). Written informed consent to participate in this study was provided by the participants' legal guardian/next of kin.

## Author Contributions

Conceptualization: S-HT, G-BC, and K-LL. Data curation: I-HL and Y-TS. Methodology: C-HC and Y-JC. Resources: K-LL. Supervision: C-HC and K-LL. Writing—original draft: S-HT and G-BC. Writing—review and editing: I-HL and Y-JC. All authors contributed to the article and approved the submitted version.

## Conflict of Interest

The authors declare that the research was conducted in the absence of any commercial or financial relationships that could be construed as a potential conflict of interest.

## Abbreviations

CPET: cardiopulmonary exercise testing
peak VO2: peak oxygen consumption
AAM: age at menarche
VBIA: vector bioelectrical impedance analysis
BMI: body mass index
FMI: fat mass index
FFMI: fat-free mass index
MET: metabolic equivalent
AT: anaerobic threshold
peak PD: peak VO2 to predicted value.
